# Editorial: Understanding anti-trypanosomatid immune responses: The key to developing protective strategies against them

**DOI:** 10.3389/fimmu.2022.993315

**Published:** 2022-09-21

**Authors:** Cazorla S. I., Alonso-Padilla Julio, W. De Souza, Padilla A. M.

**Affiliations:** ^1^ Centro de Referencia para Lactobacilos – CERELA (CONICET), Tucumán, Argentina; ^2^ Cátedra de Inmunología, Facultad de Bioquímica, Química y Farmacia, Universidad Nacional de Tucumán, Tucumán, Argentina; ^3^ Instituto Salud Global de Barcelona (ISGlobal), Barcelona, Spain; ^4^ CIBERINFEC, ISCIII—CIBER de Enfermedades Infecciosas, Instituto de Salud Carlos III, Madrid, Spain; ^5^ Instituto de Biofísica Carlos Chagas Filho, Federal University of Rio de Janeiro, Rio de Janeiro, Brazil; ^6^ CMABio, Universidade do Estado do Amazonas-UEA, Manaus, Amazonas, Brazil; ^7^ Center for Tropical and Emerging Global Diseases, University of Georgia, Athens, GA, United States

**Keywords:** neglected tropical disease, kinetoplastid microorganisms, immune response, immunomodulation, trypanosomatids

Trypanosomatid parasites are kinetoplastid microorganisms, some of which cycle between the gut of insect vectors and the tissues of vertebrate hosts (1). In the vertebrate hosts, these parasites alter the expression of virulence genes and modify biological and antigenic properties in order to counteract the host immune responses and establish a persistent infection (2, 3).

The diseases caused by trypanosomatids are among the so-called neglected tropical diseases (NTDs), which affect millions of individuals mostly in low and middle-income countries from tropical and subtropical regions. In addition to their close relationship with poverty, these diseases permanently deform and disable a large number of poor people, who are thus trapped in their poverty situation. Sadly, these diseases are often forgotten by governments and the pharmaceutical industry, due to the lack of political voice of the affected populations and the reduced economic revenue from this low-income market (4, 5).

Although chemotherapy is critically important in reducing the parasite burden, the variable efficacy of the drugs against different clinical stages of the infections, their frequently associated adverse effects, and our poor understanding of drug-resistant parasite phenotypes remain major concerns. To date, there are no licensed human vaccines against trypanosomatid infections and the majority of the immunization studies have been conducted only at the pre-clinical level (6). Thus, there is an urgent need for the development of prophylactic and/or therapeutic vaccines able to elicit an effective immune response against these parasites (7).

Trypanosomatid parasites drive major modifications of the immune system and frequently the immune response evoked does not result in protection (8). Even worse, this response might sometimes be responsible for immunopathological disorders (9). Therefore, a deep knowledge of the strategies displayed by these parasites and the response of the hosts to the infection is fundamental for the rational design of protective tools. This Research Topic focuses on the search for new prophylactic or therapeutic immunization platforms, the elucidation of the immune mechanism contributing to protection, and the strategies by which these kinetoplastids evade the host immune response.

In recent years, in addition to the identification of vaccine antigens, further efforts have focused on the development of efficient antigen delivery systems, adjuvants and vaccination regimens to enhance the protective responses to defined immunogens (10, 11).

A strong inflammatory immune response with IFN-gamma as a key cytokine is considered to be crucial in the control of *Trypanosoma cruzi* infection. However, this inflammatory profile is also frequently linked to tissue damage and considered partially responsible for the pathology of Chagas disease. Silva et al. address both aspects of the immune response by pre-treating animals with alpha-tocopherol, an isomer of the E-vitamin, prior to the infection. Alpha-tocopherol has previously been described as a potential adjuvant to enhance immune responses to vaccines in several models. They showed that a pre-treatment with several 100 mg/kg doses of alpha-tocopherol induced an increase in the number of IFN-gamma-producing immune cells and effector memory T cells, resulting in better control of parasite levels in the blood. Interestingly, this pre-treatment also resulted in reduced tissue damage associated with infection and a higher survival rate. This pathology protective effect may be due to the development of more IL-10-producing CD8^+^ T cells in the treated animals, which would counterbalance the inflammatory response while preventing an excessive destruction of the tissues. These complementary characteristics modulating the immune response make alpha-tocopherol treatment an interesting component to be tested as a vaccine adjuvant. As noted by the authors, although its use as a prophylactic measure before infection may not be practical, this treatment might become important in the context of a therapeutic vaccine, where the goal is to potentiate an already present immune response against the chronic infection while preventing further pathology. If this dual effect is also confirmed in the chronic model, treatment with alpha-tocopherol could give researchers a new tool to enhance the host response to promote complete *T. cruzi* clearance of chronic infections.

Many species and subspecies of *Leishmania* infect humans and other mammals causing a wide spectrum of diseases, ranging from cutaneous leishmaniasis (CL), mucocutaneous leishmaniasis (MCL), diffuse cutaneous leishmaniasis (DCL), and visceral leishmaniasis (VL), depending on parasite virulence factors and the immune response established by the host (12). In the Old World, VL is mainly caused by *L. donovani* and *L. infantum*, while in the New World, *L. infantum* is the primary species reported (12, 13).

A delicate balance between inflammatory and regulatory responses is required to achieve immune control of *L. infantum* (14). Furthermore, intracellular pathogens can modulate or hijack host gene expression processes through non-coding RNA-mediated regulatory mechanisms as an additional strategy to dampen the host immune response (15, 16). To gain a deeper understanding of the immune response mounted against *Leishmania* infection, Sanz et al. performed transcriptome sequencing of lymph node aspirates from dogs naturally infected with *L. infantum*. Dogs are the main domestic reservoir for visceral leishmaniasis and parasites can be found in the lymph nodes of infected dogs. They identified 5,461 differentially expressed genes (DEGs) in infected dogs compared to healthy controls. Through weighted gene co-expression network analysis, they identified four main co-expression modules associated with cell cycle processes, endoplasmic reticulum stress, regulation of the immune response, and regulation of the B cell apoptotic process. Interestingly, these co-expression modules, were correlated with the monocyte concentration in blood and the clinical stage of the sick dogs. As expected for infected animals, some of the genes displaying the highest differential expression as well as two of the four co-expression modules, were involved in the immune response. Notably, the analysis also identified 21 differentially expressed lncRNAs and one module associated with chromatin organization in sick dogs, from which the authors suggest a possible role for epigenetic regulation processes in the immunopathogenesis of canine leishmaniasis.

In recent years, an increasing number of alarming *L. donovani* cases with unusual cutaneous manifestations have been described in Sri Lanka, Nepal, and India (17, 18) Accordingly, Thakur et al. describe a systemic-immune profile of the cytokines and IgG antibodies circulating in 20 atypical cutaneous leishmaniasis patients and 18 individuals with typical visceral presentation of *L. donovani* infection from north and northeast endemic areas of India. These atypical cutaneous cases are caused by infection with *L. donovani*, a parasite whose infection usually results in a visceral systemic pathology. Authors describe a cytokine profile in the atypical cutaneous patients that more closely resembles that seen in classical cutaneous leishmaniasis cases. The cytokine profile of the studied patients displays a higher ratio IFN-gamma/IL-10, which could be associated with a better restriction of the parasites to the cutaneous environment and the prevention of visceralization. Similarly, this relatively higher level of IFN-gamma could favor the tissue damage observed in skin lesions. This profile in atypical cases differs from what is usually observed in cases of visceral leishmaniasis, despite the fact that both pathologies are the result of an infection by the same agent. As suggested by the authors, changes in the antibody pools displayed by different isolates of *L. donovani* might be in part responsible for the different outcomes, although further parasitological and deeper immunological studies are needed to test this hypothesis.

The host innate immune system plays a pivotal role in the recognition of kinetoplastid infections. Polymorphonuclear neutrophils react against protozoan parasites by different effector mechanisms, which include the release of immunomodulatory molecules (19, 20), phagocytosis, production of reactive oxygen species (ROS), and the release of neutrophil extracellular traps (NETs) (21). NETs are extracellular reticular fibrillar structures composed of DNA, histones, granulins and cytoplasmic proteins, delivered externally by neutrophils in response to different stimuli such as microorganisms, cytokines and host molecules. NET formation has been extensively demonstrated to trap, immobilize, inactivate, and kill, invading microorganisms and acts as an innate response against pathogenic invasion. Recent studies have demonstrated that the lipophosphoglycan (LPG) extracted from the surface of *T. brucei* induces the release of NETs in a time- and concentration-dependent manner (21, 22). To determine the possible pathways involved in NET formation after *T. brucei*’s LPG exposure, Zhang et al., used a combination of blocking antibodies and protein kinase inhibitors. Their work describes an activation pathway in which *T. brucei*-derived LPG induces the phosphorylation of the c-Jun N-terminal kinase (JNK) through TLR2 and TLR4 receptors on the surface of neutrophils. This JNK phosphorylation triggers the release of DNA and the burst of ROS by neutrophils resulting in the formation of the NETs. As such, this study identifies a *T. brucei*–LPG-induced activation pathway for NET formation with features that are shared by other trypanosomatid infections.

In conclusion, this Research Topic provides new knowledge regarding the host- pathogen relationships of kinetoplastid parasites and the description of potential targets and approaches that could augment protective immune responses. Some of the mechanisms that parasites can orchestrate to counteract the host defenses and establish a functional infection are also considered. Nevertheless, our understanding of the immune system interaction with these highly complex pathogens is far from complete, and many challenges remain in our fight against these parasites.

## Author contributions

All authors listed have made a substantial, direct and intellectual contribution to the work, and approved it for publication.

## Acknowledgments

The editors want to acknowledge the authors for their contributions to this Research Topic. We would also like to thank to the staff of Frontiers in Major Tropical Diseases Immunology for making it possible for us to conduct this Research Topic.

## Conflict of interest

The authors declare that the research was conducted in the absence of any commercial or financial relationships that could be construed as a potential conflict of interest.

## Publisher’s note

All claims expressed in this article are solely those of the authors and do not necessarily represent those of their affiliated organizations, or those of the publisher, the editors and the reviewers. Any product that may be evaluated in this article, or claim that may be made by its manufacturer, is not guaranteed or endorsed by the publisher.
